# The SHIELD Project: Designing an Intervention for Social Media With Young People

**DOI:** 10.1192/bjo.2022.181

**Published:** 2022-06-20

**Authors:** Gloria Cheung, Ioana Varvari, Clare Fenton

**Affiliations:** 1 University of York, York, United Kingdom; 2 Tees, Esk and Wear Valleys NHS Foundation Trust, Harrogate, United Kingdom

## Abstract

**Aims:**

The primary aims of our study is to gather ideas from young people about developing an intervention for children who first started using social media. Our study also aims to investigate whether different types of social media use are associated with impact of social media on emotions and self-esteem.

**Methods:**

An anonymous questionnaire was distributed to young people (16–25 years old), who were UK residents, through word of mouth, social media and university newsletters. We assessed participants’ baseline characteristics, including types of social media use (active, active-passive and passive), impact of social media. We also explored young people's idea on developing a social media intervention, including how it should be delivered, topics that needs to be covered. Quantitative data were analysed using descriptive statistics and ordinal regression analysis.

**Results:**

90 young people completed the questionnaire. 37.8% of the participants started using social media before 13 years old. Analysis has shown that interacting with other users and creating social media content is associated with higher self-rated negative impact on self-esteem from social media, but there is no association between impact on self-esteem and reacting to other's social media content or browsing other's social media content. Types of social media use were not associated with a self-rated impact of social media on emotions. Regarding the co-development of an intervention for social media, young people believe the best ways to distribute information about social media is through an interactive session by professionals (36.7%) or teaching it in class (28.9%) while the least popular ways are poster/booklet (1.11%) and mobile phone app (1.11%). The majority of young people felt the following topics on social media to be useful to cover during interventions, including risks on social media (85.6%), emotional safety on social media (81.1%), social media hygiene (70.0%), coping strategies and finding help on social media (66.7%), communication on social media (58.9%).

**Conclusion:**

Although types of social media use are not associated with impact on emotions from social media, those who create social media content are at higher risk of having more impact on self-esteem. Interventions should be developed to help protect or improve self-esteem while using social media. This could be done by focusing on different topics. Future interventions for young social media users should be interactive and led by experts. They should also start before children reaches the common legal age of social media use to make them more resilient to the digital world.

